# The Interactive Child Distress Screener: Development and Preliminary Feasibility Testing

**DOI:** 10.2196/mhealth.9456

**Published:** 2018-04-19

**Authors:** Sonja March, Jamin Day, Kirsty Zieschank, Michael Ireland

**Affiliations:** ^1^ Institute for Resilient Regions University of Southern Queensland Springfield Central Australia; ^2^ School of Psychology and Counselling University of Southern Queensland Ipswich Australia

**Keywords:** child, preschool, mental health, symptom assessment, self-assessment (psychology)

## Abstract

**Background:**

Early identification of child emotional and behavioral concerns is essential for the prevention of mental health problems; however, few suitable child-reported screening measures are available. Digital tools offer an exciting opportunity for obtaining clinical information from the child’s perspective.

**Objective:**

The aim of this study was to describe the initial development and pilot testing of the Interactive Child Distress Screener (ICDS). The ICDS is a Web-based screening instrument for the early identification of emotional and behavioral problems in children aged between 5 and 12 years.

**Methods:**

This paper utilized a mixed-methods approach to (1) develop and refine item content using an expert review process (study 1) and (2) develop and refine prototype animations and an app interface using codesign with child users (study 2). Study 1 involved an iterative process that comprised the following four steps: (1) the initial development of target constructs, (2) preliminary content validation (face validity, item importance, and suitability for animation) from an expert panel of researchers and psychologists (N=9), (3) item refinement, and (4) a follow-up validation with the same expert panel. Study 2 also comprised four steps, which are as follows: (1) the development of prototype animations, (2) the development of the app interface and a response format, (3) child interviews to determine feasibility and obtain feedback, and (4) refinement of animations and interface. Cognitive interviews were conducted with 18 children aged between 4 and 12 years who tested 3 prototype animated items. Children were asked to describe the target behavior, how well the animations captured the intended behavior, and provide suggestions for improvement. Their ability to understand the wording of instructions was also assessed, as well as the general acceptability of character and sound design.

**Results:**

In study 1, a revised list of 15 constructs was generated from the first and second round of expert feedback. These were rated highly in terms of importance (mean 6.32, SD 0.42) and perceived compatibility of items (mean 6.41, SD 0.45) on a 7-point scale. In study 2, overall feedback regarding the character design and sounds was positive. Children’s ability to understand intended behaviors varied according to target items, and feedback highlighted key objectives for improvements such as adding contextual cues or improving character detail. These design changes were incorporated through an iterative process, with examples presented.

**Conclusions:**

The ICDS has potential to obtain clinical information from the child’s perspective that may otherwise be overlooked. If effective, the ICDS will provide a quick, engaging, and easy-to-use screener that can be utilized in routine care settings. This project highlights the importance of involving an expert review and user codesign in the development of digital assessment tools for children.

## Introduction

### Background

Behavioral and emotional problems are among the most common reported mental health difficulties in children younger than 12 years of age [1-3]. Such problems can interfere with a child’s social and academic functioning and increase the risk of developing more severe problems such as depression, anxiety, and behavioral disorders [3-5]. As early intervention can alter the trajectory of disorder development and minimize the social, emotional, and economic burden of mental illness [3,6], universal screening for early identification is important. Dowdy et al [7] advocate a population-based approach to monitoring and addressing mental health difficulties in school-aged children, with universal screening as the first step in a multistage gating system. To this end, recommendations (eg, [8]) suggest that childhood screening instruments should meet 3 goals: (1) ability to identify behaviors that are known risk factors for further behavioral and emotional difficulties; (2) facilitate a timely assessment of children in an inexpensive manner; and (3) identify children at-risk and in need of further assessment, support, or intervention (ie, adequate specificity and sensitivity).

The assessment of general behavioral and emotional difficulties in children routinely relies on reports from parents, caregivers, and education professionals. Although information from these key informants is important, child self-report is a valuable source of clinical information that is often overlooked. Additionally, few self-report screening instruments exist that are suitable for primary school-aged children, particularly universal screeners with a focus on early detection and prevention. For example, in a recent review of instruments for children and adolescents, Deighton et al [9] identified only 11 instruments that included a self-report component, with only 8 of these suitable for children younger than 12 years, and 5 suitable for children aged 10 years and younger. Furthermore, of those measures suitable for younger ages, only 2 could be considered brief screeners, or containing fewer than 30 items—the KIDSCREEN 10 and 27-item versions [10] and the 25-item Youth Rating Scale from the Behavioral and Emotional Rating Scale-2 ([11]). Additionally, the Behavioral and Emotional Rating Scale is not free for research or clinical use and the European KIDSCREEN provides an index of health-related quality of life rather than emotional and behavioral difficulties, and to our knowledge is not suitable for use with children younger than 8 years of age.

Screening directly with children may facilitate quick identification of a range of social and behavioral indicators [9], while also having the potential to capture internalizing (emotional) difficulties that parents or caregivers have not been able to observe [12]. However, there are numerous administrative challenges that may preclude children from responding or impact the reliability of the information collected [13,14]. Children may find the traditional text-based rating scales difficult because of their limited attention spans or difficulties with reading, language, and item comprehension [13,14], with these issues more pronounced in younger children (eg, 5-8 years). In addition, given there are significant developmental variations between the ages of 5 and 12 years, crafting items using appropriate language that is broadly suitable across various ages is a challenge for scale developers.

Despite this, researchers have demonstrated that when age-appropriate methods are used, valid and reliable self-report information can be obtained from children even as young as 5 or 6 years (eg, [13]). Much of this work has focused on adjusting the delivery modality (eg, clinical interviews), using novel stimuli (eg, interacting with puppets), or enhancing the traditional response scales with pictorial elements [15-18]. More recently, engaging and innovative approaches using digital technologies have been trialed. Examples include the Dominic Interactive [19], a computer-based diagnostic assessment that utilizes child-friendly (static) images, and maps onto 7 Diagnostic and Statistical Manual of Mental Disorders, Fourth Edition disorders with demonstrated reliability and construct validity [20]; the Mood Assessment via Animated Characters [21], which uses digitally animated characters to assess internalized mood states (feelings) in young children aged between 4 and 11 years, and which has been shown to discriminate between anxious and nonanxious children [14]; and TickiT, a psychosocial screening app for adolescent youths that has been employed in hospital settings [22,23]. Similarly, a computer-administered, pictorial version of the Strengths and Difficulties Questionnaire (SDQ) has also been investigated with children aged between 8 and 15 years, with some evidence of clinical sensitivity (in children 11 years and older), higher user satisfaction ratings, and improved engagement compared with the standard pencil-and-paper version [24]. These efforts support the feasibility of digital assessment tools for children; however to our knowledge there are currently no digitally delivered, universal self-report screeners for emotional and behavioral difficulties that are suitable for primary-school children aged 5 to 12 years. There is also very little information available about how the aforementioned instruments were developed and which components were demonstrated to be effective.

### Objectives

This research describes the development process of the Interactive Child Distress Screener (ICDS), a new Web-based screening instrument for early identification of emotional and behavioral problems. The ICDS is designed to be easily administered within community settings, such as general practitioner clinics or education contexts, using modern touchscreen devices that are ubiquitous and familiar to most school-age children (eg, tablets and mobile phones), and with potential to facilitate rapid feedback to those administering the instrument (eg, educators or primary care professionals) through automated scoring. The ICDS differs from the aforementioned digital instruments in that it aims to provide brief, universal screening for general behavioral difficulties and emotional distress (nondiagnostic) and utilizes short, animated cartoons in place of text-based items to convey common childhood difficulties in a way that is familiar, engaging, and relatable for even young children (eg, 5-6 years) while also remaining appealing to children in later primary-school (eg, 10-12 years).

To maximize the potential effectiveness of the ICDS, a thorough initial development and feasibility-testing process was implemented and reported here. The development process utilized a mixed-methods approach incorporating expert review to formulate and develop item content, along with user involvement from children to develop and evaluate the response format and working prototypes of both animations and user interface. Item content development and refinement is described in study 1, followed by animation and interface development and refinement which is described together in study 2. Lessons learned from this approach are presented in the Discussion.

## Methods

### Study 1: Item Content Development and Refinement

Content for the ICDS animations was drawn from 3 existing validated instruments used to assess general distress in children. We initially selected 2 parent-report measures—the SDQ [25] and the Child Behavior Checklist (CBCL) [26] —because they are frequently cited and widely used instruments for assessing general behavioral and emotional difficulties in children [27,28]. However, the CBCL is a longer instrument used primarily as a broad comprehensive assessment tool. Thus, we utilized its brief counterpart, the Brief Problem Monitor (BPM) [29], which includes original items from the CBCL. Further review of the literature revealed a brief, 16-item child self-report instrument, the Me and My School Questionnaire (M&MS) [30]. This scale has been validated with children as young as 8 years (ie, year 4 students) as well as in clinical and nonclinical samples [31] and has demonstrated a clear 2-factor structure (behavioral and emotional problems) and adequate internal consistency with both year 4 and year 7 students.

The initial task of developing a suite of animations representing children’s behavioral and emotional difficulties required that we first identify key item groupings that best represent the primary constructs or domains covered by the validated instruments. Our aim was to identify a thematically common set of domains that had proved useful in previous screening instruments. Before proceeding to the animation development phase, there were 3 issues to consider regarding the content validity of the item groups and which formed the focus of study 1. The first consideration was whether our proposed item groupings (constructs) included items that were similar enough to each other to plausibly tap a global distress construct and to check whether item subgroupings were plausible. The second consideration was whether our proposed item groupings had potential to be depicted clearly through the use of brief animations, such that the target difficulty would be understood by primary school-age children. Relatedly, it was important to evaluate whether this was likely to be feasible using a single animation or whether multiple animations would be required. The third consideration was to identify the relative importance of each construct for inclusion in a broad screener of general behavioral and emotional difficulties in children.

To address these considerations, we sought a review of our proposed construct groupings from a small panel of experts, broadly adopting a strategy outlined by Kassam-Adams et al [32] regarding assessment of content validity through expert panel review. Though these guidelines were provided for evaluation of eHealth interventions, their approach to systematically assessing the relevance, effectiveness, and appropriateness of activity-target pairings (ie, item-construct pairings in our study) through mixed quantitative and qualitative expert responses was translatable to this study.

Item content development followed an iterative process and consisted of four steps, which are as follows: (1) initial construct development, (2) preliminary expert content validation, (3) item refinement, and (4) final expert validation.

#### Step 1: Initial Construct Development

We first collated all 60 items from the SDQ, BPM, and M&MS into an item pool. As there was a significant amount of overlap among items, these were grouped together by the first and second authors (both psychologists with prior experience in assessment with children) according to common themes. Some items had clear conceptual overlap (eg, “I am unhappy” from the M&MS and “Often unhappy, depressed or tearful” from the SDQ). Some items described related but potentially discrete problems (eg, the M&MS contains 2 sleep-related items: “I have problems sleeping” and “I wake up in the night”) that we thought would be difficult to distinguish from each other through brief animations and thus were grouped together (eg, using a broader category of “sleep difficulties”). Differences in item phrasing arising from self-report (first person) versus parent-report measures (third person) were not considered relevant to this process as it was peripheral to our goal of identifying common themes indicative of behavioral and emotional difficulties in children. The outcomes of step 1 are presented in the Results section and Multimedia Appendix 1.

#### Step 2: Initial Expert Review of Constructs

In step 2, we sourced 9 panel respondents (78%, 7/9 female) among the professional networks of the researchers. Respondents were invited based on identified clinical experience working with children (89%, 8/9), methodological expertise in the area of clinical research (67%, 6/9), or psychological assessment and measure validation (67%, 6/9), with some participants reporting expertise across multiple areas. Overall, the median level of experience across participants was 17 years in their respective fields (range, 7-35 years).

Respondents were presented with the list of 14 proposed domains along with the individual items that were grouped to form this construct (ranging from 2-8 items, as shown in Multimedia Appendix 1). For each grouping, respondents answered 3 questions using a 7-point Likert scale: (1) importance: “How important is the construct for inclusion in a brief screener of general emotional and behavioral difficulties in children?” (1=Not at all important to 7=Extremely important); (2) conceptual consistency: “How well do the individual items hang together as a common theme or construct?” (1=Very poorly to 7=Very well); and (3) identifiability: “How likely is it that a child could identify this behavior or difficulty if depicted in an animation?” (1=Very unlikely to 7=Very likely). An open-ended text box was included after each conceptual group to allow respondents to provide a rationale for their ratings or any further reflections on the items or our proposed item groupings (eg, whether a group of items should be separated into 2 constructs).

#### Step 3: Refinement of Item Content

In step 3, expert ratings and feedback were reviewed by the research team with special attention given to those domains that had the poorest ratings in any of the 3 categories (≥ 1 SD below the overall mean). Qualitative feedback was also reviewed carefully for further insight. Constructs that had low conceptual consistency or were discussed qualitatively as not “fitting” together well were candidates for division into multiple constructs or for the removal of some items from one construct to be merged into another. Item groupings with lower ratings of importance, or viewed as likely to be especially difficult to depict using animations, were considered for removal. Decisions were data-informed and based on clinical relevance using an iterative process where changes were continually reviewed by the research team. This step produced a refined list of item groupings.

#### Step 4: Follow-Up Expert Review of Constructs

In step 4, a follow-up expert review process was utilized to collect feedback on the refined list of item-construct groupings. Of 9 participants included in this study, 8 provided feedback at step 2 (75% female; 6/8); median years of experience in field was 17; clinical experience working with children: 88% (7/8); methodological expertise in the area of clinical psychology research: 75% (6/8); and psychological assessment and measure validation: 63% (5/8).

The procedure was similar to the first expert panel survey. Using the same scale, respondents provided ratings for the perceived importance of the refined constructs (see Multimedia Appendix 1), as well as face validity of the internal consistency (ie, how well items “hang” together). A separate section was included containing the list of constructs that had been removed following the first round of feedback (Nervous, Low self-worth or self-esteem, Internalizing, Illicit or covert behaviors, Immature, Impulsive behavior, and Caring or helpful), with respondents asked to rate their importance for inclusion. Respondents were not asked to rate the potential identifiability of constructs through animation as this was evaluated more directly through interviews with children using prototype animations (study 2).

### Study 2: Animation and Interface Development and Refinement

In addition to the initial expert review to confirm importance, conceptual consistency, and identifiability of items in study 1, we also conducted research with children to test and refine sample animations and the app interface. The aim was to determine whether the intended meaning of pilot animations could be accurately identified; whether the response instructions were understood; and whether the characters, sounds, and animation style were acceptable and engaging. Similar “codesign” approaches have been used successfully in the development of innovative eHealth and mHealth technologies, where prospective users are involved collaboratively during design and development stages to provide valuable feedback and direction for ongoing development (eg, [22,23]).

This study consisted of four steps, which are as follows: (1) development of prototype animations, (2) development of the interface and response format, (3) child interviews to determine feasibility and obtain feedback, and (4) refinement of animations and interface.

#### Step 1: Development of Prototype Animations

From the 15 revised constructs produced in study 1, the 3 following items were selected for development of prototype animations: (1) Sad or depressed, (2) Worried, and (3) Sleep problems. The constructs Sad or depressed and Worried were selected based on expert ratings of high importance, whereas sleep problems was considered particularly amenable to animation and provided us with a broader coverage of content areas for piloting with the respondent group.

**Figure 1:**
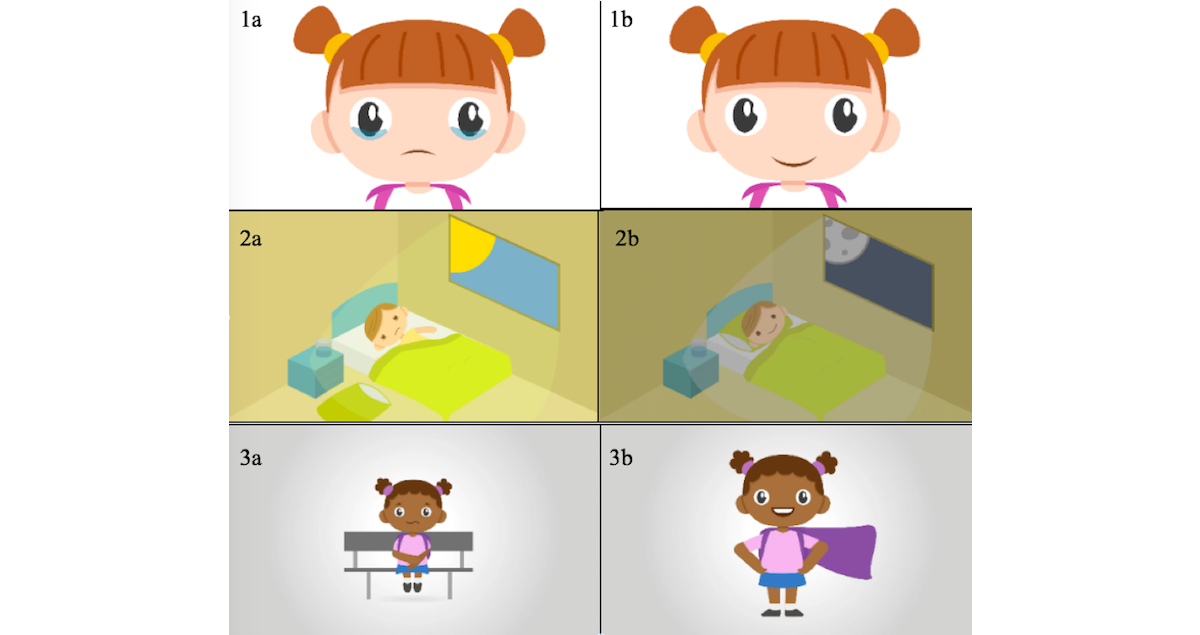
Screenshots from early versions of the pilot animations. Images 1a and 1b show paired animations for the construct Sad or Depressed. Images 2a and 2b reflect the construct Sleep Problems. Images 3a and 3b reflect the construct Worried.

For each construct, 2 prototype animations were developed; 1 “negative” animation showing a child experiencing the difficulty described by that construct and its candidate items (see Figure 1), and one “positive” animation indicating the absence of that difficulty or showing a child demonstrating a contrasting (ie, positive) behavior. This resulted in 6 pilot animations labeled: 1S (Sad) and 2H (Happy), 3SP (Sleep Poorly) and 4SW (Sleep Well), and 5W (Worried) and 6C (Not Worried). The rationale for the response format choice is explained below at step 2.

Animations were developed in consultation with an animator and a graphic designer. To encourage engagement, animations were designed to be brief (eg, 6-10 s), with each demonstrating a short, focused scenario showing either the positive or negative depiction of the intended construct. A mix of genders and ethnicities was used for the characters, but the same character was used in each animation pair for consistency and to minimize distraction. Stylistically, characters featured in the animations resembled cartoon children, which are easily relatable. Characters were given simple features with large eyes for expressiveness, and warm, bright colors for clothes and backgrounds. Contextual features were kept to a minimum so that children would not be distracted by nonessential information and so the target item was not specific to a context, with background objects only included if they enhanced the intended message (eg, an alarm clock and bed for the Sleep problems videos).

As a first step, the research team generated ideas for animating the item content based on common characteristics identified from the pooled items for each construct. A suggested storyboard was created for each animation detailing (1) the character’s actions, (2) the scenery and objects to include or for the character to interact with, (3) sound effects that might enhance the message (eg, sound of a child crying), and (4) colors and other special effects that might further convey the construct’s meaning.

Storyboards were then shared with the animator, who prepared a first pass of animations that was reviewed by the research team. This iterative process continued for each animation until both parties were satisfied with the pilot version. Outcomes from step 1 are provided in the Results section.

#### Step 2: Development of the Interface and Response Format

Development of the app and its interface required consideration of multiple factors, including (1) the response format, (2) the technology stack (eg, Web-based vs native app), (3) layout and colors, and (4) audio versus text-based instructions.

##### Response Format

We chose to develop a 2-stage response format in light of research suggesting younger children typically tend to respond at the extreme ends of rating scales and may perform better with dichotomous, forced-choice responses (eg, [33]). The app was developed such that after viewing both animations, children are asked to select one animation in response to the question “Which one is more like you?” To provide additional information, we also included a second follow-up question: “How much is this like you?” where children were asked to select either “A lot like me” or “A little like me.” The aim of this approach was to present children with simple dichotomous response options while maximizing the range of potential variability in response scores (ie, 1-4 for each item rather than binary responses). To our knowledge, the validity of such a response format has not yet been tested within digital screening instruments. As such, we decided to pilot this approach given that it would be trivial to later eliminate the second response stage, if it proved too complex or unreliable during administration.

##### Technology Stack

Although native apps written using a platform-specific code (eg, Swift for iOS, Java for Android) are typically considered to have some advantages over Web-based apps in terms of speed and access to in-built device functions, for the pilot version of the ICDS we decided to harness the capabilities of modern Web-based technologies (eg, HTML5, JavaScript, and a responsive design) to ensure widespread accessibility. A PHP: hypertext preprocessor backend based on the open-source WordPress framework was utilized for administrative access, with data collected in a structured query language (SQL) database stored on a secure server within the host university’s research infrastructure. Using this combination of technologies, the app was enabled to be viewed through the Web-browser on any modern smart-device (eg, phone, tablet, personal computer), making it highly compatible and transferrable across testing scenarios.

##### Interface (Layout, Colors, and Instructions)

An iterative and collaborative development process involving the research team, the Web developer, and the graphic designer was utilized to develop an early working prototype of the app interface. The flow of the initial version of the app was developed as follows. Children are asked to select an avatar (or “buddy”) to accompany them through the app and then provide basic demographic information (age and gender). The following screens contain an animation pair (ie, one “item”), with both animations (positive and negative) presented side-by-side in a randomized order. Children play the highlighted video first, followed by the second video that is only available to view after the first animation finishes. Upon the completion of the second animation, verbal instructions commence asking children to answer the question “Which one is most like you?” For the first 2 items, written instructions are also displayed while being spoken by the child’s selected avatar that appears at the bottom of the screen. For subsequent items, instructions are not spoken or written unless the child taps the buddy helper for assistance. After providing a response, the app automatically advances to the next item.

The interface was developed using a simple, clean design and a bold, bright color palette. The suite of avatars (buddies) introduced at the beginning of the screening instrument was designed to promote engagement and facilitate understanding and use of the app. Examples of the resulting interface are provided in the Results section.

#### Step 3: Qualitative Child Interviews

Step 3 adopted a cognitive interviewing approach [34] to obtain feedback from children regarding the interpretability and acceptability of the animations, instructions, and response format. A convenience sample of children was recruited through personal networks. Eighteen children (10 females) aged between 4 and 12 years participated in the interviews. Of the 18 participants, 2 were “British Caucasian,” 3 were “South-East Asian (Philippines),” 2 were “New Zealand Caucasian,” and 11 were “Australian Caucasian.” Most ages were represented by at least 1 male and female (see Table 2 in Results). Qualitative data reached saturation at N=18 and therefore, we determined that sufficient information for refining and improving prototype animations had been gathered.

Both the child and a parent were required to provide consent to participate in the interviews that lasted between 15 and 30 min, with children permitted as much time as they required to answer all questions. Questions were asked verbally, with answers recorded verbatim by the interviewer along with other relevant descriptive information about the child’s demeanor or nonverbal responses (eg, “child shrugged” to indicate lack of understanding of the item). The interviews included questions that checked children’s understanding of animated items, understanding of instructions and response format, and acceptability of the prototype app.

##### Understanding of Animated Items

Children were first shown each animation and asked “What do you think is happening for the boy or girl in this video?” Responses were noted and the interviewer made a judgment on the “correctness” of the response (ie, whether the child’s response matched the intended behavior that was being animated). If the child’s response was considered incorrect, the intended meaning of the animation was provided by the interviewer. Children were then asked to rate the pilot animation on how well they thought it captured the intended behavior, using a colored, cartoon visual analog scale from 1 to 5 (1=“NO! I HATE it. Change it completely”; 3=“OK. I kind of like it”; 5=“YES! I love it. It’s exactly right”). Children were then prompted to provide a “better” way of showing the intended target problem and whether they could think of a time when they (or someone they knew) felt the same as the character. The latter question was intended to determine how well children were able to relate the behaviors shown in the animations to their own experiences.

##### Understanding of Instructions and Response Format

To evaluate the instructions and response format, children were asked if they could explain what was meant by the instruction “which video is most like you?” We then asked children to describe what it means if the character is “a lot like you” or “a little like you.”

##### Acceptability

Finally, we obtained general acceptability ratings of the characters, animation style, and sounds using a mix of open-ended verbal feedback (ie, “what did you like”, “what didn’t you like”) and quantitative ratings using the pictorial Likert scale described earlier (ie, “show me on the chart how much you liked it”). At the end of the interview, participants were also asked an open-ended question as to whether they had any other ideas that would make the animations easier to understand. Feedback from the child interviews regarding the characters, sounds, and behaviors depicted in the animations was collated and reviewed by the research team.

#### Step 4: Refinement of Animations

In step 4, we focused particularly on feedback for the animations that were misinterpreted or not well-understood, along with suggestions from children that might help to improve the interpretability or likeability of animations in general. Suggestions for enhancing facial features and emotional expressiveness, along with increasing the contrast between paired animations, were deemed particularly important. In response to the feedback obtained, we developed new storyboard outlines to target the identified deficits in understanding and worked with the animator to implement these changes. Changes primarily included increasing the expressiveness of characters such as adding emphasis to the character’s eyes to make them twinkle or fill with tears, more exaggerated mouth movements, and adding eyebrows to enhance expression. Other details were also added to improve the interpretability of the intended behavior such as beads of sweat, tousled hair, and blinking eyes, whereas additional sounds and movement were incorporated such as giggling, crying, or shoulder movements to accentuate body language for laughing or sobbing.

Further context was also added by including new objects or symbols such as “thought bubbles,” a dream bubble of a jumping sheep, shadow creatures to represent a nightmare, an alarm clock with a grumpy face, and lightning bolts, as well as butterflies in the stomach area to represent worried, and a red heart shape beating quickly with sound effects. These revisions resulted in 3 new pairs of animations.

## Results

### Study 1: Item Content Development and Refinement

#### Step 1: Initial Construct Development

In Step 1, we conducted a preliminary review and grouping of the 60 items from the SDQ, BPM, and M&MS instruments. This resulted in the identification of 14 domains, which are outlined in Multimedia Appendix 1.

#### Step 2: Initial Expert Review of Constructs

Mean ratings of perceived importance, internal conceptual consistency, and interpretability provided by panel experts were computed for each of the initial domains are shown in Table 1, sorted in order of importance for inclusion in a screening tool as rated by the panel. The panel considered areas pertaining to feeling sad or depressed, nervous or shy in social settings, and worried or anxious as being most important for a screener of general difficulties in children, followed by noncompliance and aggressive behavior problems. The panel also suggested that impulsive and inattentive behavior might be the most difficult areas for children to identify through animations.

Panel members provided a number of comments relating to the constructs, with the most common feedback being that some item groupings should be split into distinct constructs. For example, items originally grouped as “worried or anxious” were considered to tap into separate domains of “worried” and “fearful.” Similarly, the “irritable, argumentative, easily loses temper” domain was seen to contain both outward, externalizing behaviors (eg, “I get very angry,” “Argues a lot”) as well as internalized behaviors (eg, “I am calm,” “Stubborn, sullen, and irritable”), which were recommended to be considered distinct.

#### Step 3: Refinement of Item Content

Analysis of expert ratings and qualitative feedback produced a refined list of item groupings in step 3 (see Multimedia Appendix 1 and Table 1). The constructs initially labeled as Impulsive behavior, Helpful and considerate of others, and Illicit or covert behavior were removed due to low importance ratings (>1 SD below mean), along with agreement within the research team that these appeared to have less relevance for a broad, universal screener. The constructs labeled as Nervous or shy, Worried or anxious, Sad or depressed, Irritable or argumentative, and Social problems were separated into multiple groupings. For example, items originally grouped as Social problems were seen as mapping onto 2 converging but distinct ideas: Difficulty making friends and Bullied or teased by other children. Some items that no longer appeared to fit within any existing constructs were removed such as “feels worthless or inferior” which was previously grouped under the Sad or depressed construct.

#### Step 4: Follow-Up Expert Review of Constructs

Results from the follow-up expert panel survey are presented in Table 1. Other than Shy (mean=5.75) and Physical symptoms (mean=5.38), all constructs had a mean importance rating of at least 6 out of 7 (overall mean=6.32, SD 0.42). One respondent commented that targeting some physical symptoms such as “sickness” may not be a good indicator of emotional difficulties in children who have chronic illness. It was decided to retain this item for testing in the full ICDS. The constructs that had been removed (not shown in the table) received the lowest mean importance ratings overall (range, 1.50-4.25; mean=2.75, SD 0.92). In terms of perceived face validity of items informing each construct, these had high overall ratings (range, 5.50-6.88, mean=6.41, SD 0.45).

### Study 2: Animation and Interface Development and Refinement

#### Step 1: Development of Prototype Animations

Still screenshots representing the early prototypes of the 6 pilot animations developed in step 1 are shown in Figure 1. As an example, for the construct Sleep problems, the first iteration of the animation showed a child tossing and turning in bed at night, unable to fall asleep, throwing his pillow on the ground, and waking up tired and grumpy the next morning with lines under the eyes and a frowning face. Its paired animation demonstrated a child yawning, falling asleep peacefully at night, and then waking up happy and refreshed in the morning when the sun rises.

#### Step 2: Interface Development

**Figure 2:**
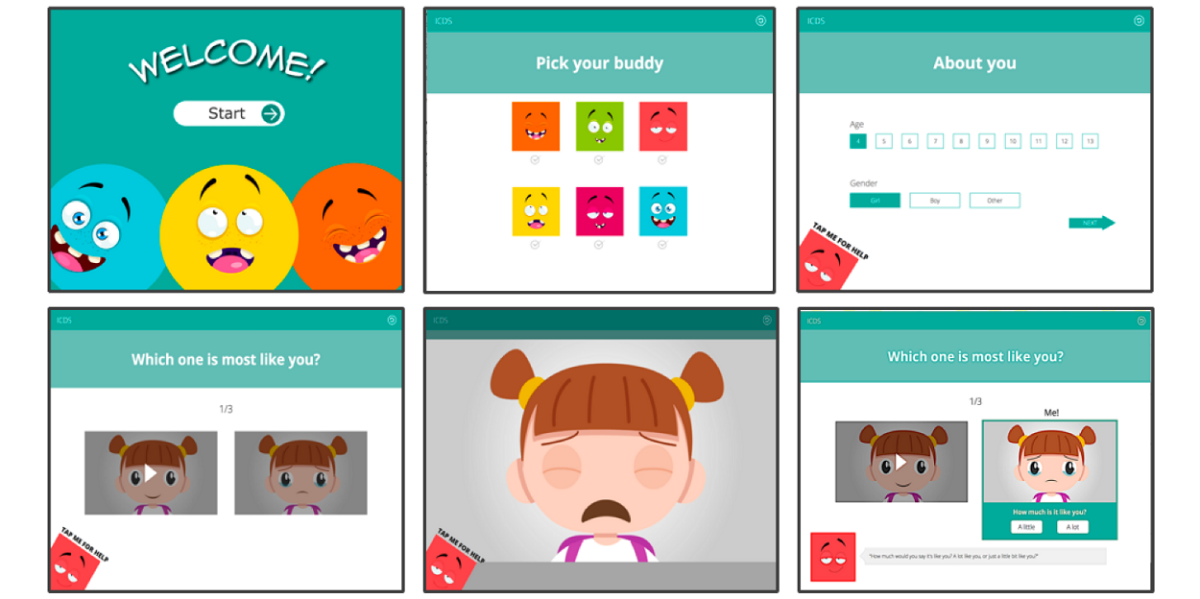
Screenshots from early prototype of the Interactive Child Distress Screener (ICDS) interface. From left to right: Top row: welcome screen, avatar (“buddy”) selection, and demographics. Bottom row: animation pairs, video pop-out, “How much is it like you?” selection with audiovisual instruction text spoken by the “buddy” helper.

Figure 2 provides example screenshots from the prototype version of the app developed in step 2. A number of revisions were made to early versions of the interface based on internal review and testing, with a particular focus on issues that might limit the use and effectiveness of the app. For example, it was noted that animated videos would be clearer if presented as full screen pop-out videos rather than side-by-side animations. Thus, the app was amended so that each animation would use the full-screen window when viewed. Timing of responses was altered so that the child could not choose the response option until both videos had been played. It also became apparent that it would be beneficial to automatically play audio instructions for the first 2 items (rather than just the first item) to help ensure children remember what they were required to do beyond the first screen. Following the second item, the interface was further adapted such that instructions could be replayed on request by tapping on the buddy helper.

#### Step 3: Qualitative Child Interviews

##### Understanding Animated Items

Table 2 summarizes the number of children considered to have correctly interpreted each of the 3 items across each age. All children were able to correctly identify happy and sad or provided a similar response (eg, “upset”). Approximately half of the children correctly identified sleeping poorly and sleeping well, with no clear age-related pattern. Correct responses to these items included comments such as “he had a good sleep,” “the boy didn’t get enough sleep,” and “slept badly,” whereas incorrect responses included comments such as “tired and sleepy” or “sad in his bed.” For the worried and not worried pair of videos, none of the younger children (<8 years) were able to respond correctly, whereas the children who were 8 years and older had more success (54%, 6 out of 11 correct for worried; 45%, 5 out of 45 correct for not worried). Younger children provided comments such as “hungry,” “just a bit sad,” and “happy”; whereas older children responded with comments such as “anxious, worried, waiting,” “alone and anxious, waiting at a bus stop,” and “confident.”

When asked to rate each video on a scale of 1 to 5 for how well it captured the intended target, children tended to rate positive videos highest, suggesting their use of the rating scale in this context may have been more reflective of how “good” or “bad” the behavior was seen to be, rather than how well our animations did at capturing that behavior. These ratings are shown in Table 2.

**Table 1:** Ratings for constructs based on initial expert panel review, ordered from most (7) to least (1) perceived importance for a screening instrument.

Domain label		Importance, mean (SD)	Hangs together^a^, mean (SD)		Identifiable, mean (SD)	
**Survey 1: Preliminary domain label**						
	Sad or Depressed	6.88 (0.35)		6.22 (1.09)		6.13 (0.35)
	Worried or Anxious	6.63 (0.74)		6.00 (1.12)		5.00 (1.20)
	Nervous or shy in social settings	6.63 (0.52)		6.11 (1.17)		4.88 (1.55)
	Noncompliant behavior	6.63 (0.74)		6.56 (0.73)		4.75 (1.49)
	Aggressive behavior	6.38 (1.06)		6.11 (1.05)		5.38 (1.30)
	Irritable or argumentative or easily loses temper	6.38 (1.19)		5.56 (1.24)		6.25 (0.71)
	Sleep problems	6.25 (1.16)		6.22 (1.3)		5.75 (1.39)
	Hyperactive behavior	5.88 (1.13)		6.44 (1.01)		5.25 (1.04)
	Inattentive behavior	5.88 (1.13)		5.78 (1.92)		3.75 (1.39)
	Destructive behavior	5.63 (1.30)		6.11 (0.78)		5.25 (1.67)
	Social problems^b^	5.60 (1.34)		4.80 (0.84)		6.60 (0.55)
	Impulsive behavior	5.13 (1.73)		4.89 (1.69)		3.75 (1.39)
	Helpful or considerate of others	5.13 (2.17)		6.44 (1.01)		5.38 (0.92)
	Illicit or covert behavior	5.00 (1.20)		4.78 (1.99)		4.75 (1.04)
**Survey 2: Refined domain label**						
	Angry	6.88 (0.35)		6.63 (0.74)		
	Sad or depressed	6.88 (0.35)		6.13 (1.13)		
	Worried	6.75 (0.46)		6.63 (0.74)		
	Fearful	6.63 (0.52)		6.50 (0.93)		
	Noncompliance (home)	6.50 (0.76)		6.75 (0.71)		
	Difficulty making friends	6.50 (0.76)		6.13 (1.36)		
	Physically aggressive	6.50 (0.53)		5.50 (1.41)		
	Noncompliance (school)	6.38 (1.06)		6.88 (0.35)		
	Argumentative	6.38 (1.06)		6.88 (0.35)		
	Bullied or teased by other children	6.25 (1.04)		6.13 (1.36)		
	Hyperactive behavior	6.00 (1.20)		6.75 (0.71)		
	Inattentive behavior	6.00 (1.20)		6.50 (0.93)		
	Sleep problems	6.00 (0.76)		5.50 (1.20)		
	Shy	5.75 (1.28)		6.75 (0.46)		
	Physical symptoms	5.38 (1.41)		6.50 (1.07)		

^a^How well individual items hang together as a common theme or construct.

^b^Four responses missing from Survey 1 for Social Problems due to technical error.

Regarding suggested changes to animations to better capture the intended behavior, most responses appeared to fall into one of 3 categories. First, some suggested changes for making the animations more exaggerated to more clearly capture the emotion (eg, “jumping up and down and looking excited”; “show him crying more”); others focused on adding more context to the videos, usually relating to a specific scenario or setting (eg “having fun on a playground”; “she can’t find her Mum and Dad”; “getting a high score in a math’s test”); whereas others suggested the addition of iconic cartoon elements with which they may be familiar from other media such as thought or dream bubbles, looking like a “zombie,” or dropping ice cream on the floor and crying. Abbreviated responses to questions regarding interpretation and ways to improve animations for each child are presented in Multimedia Appendix 2.

**Table 2:** Descriptive statistics from child interviews showing age, gender, and response characteristics for participating children.

Variable		Child age (years)									Total (n)		Mean (SD)^a^	Choice (n)^b^
		4	5	6	7	8	9	10	11	12				
**Gender (n)**														
	Male	1	1	1	0	1	1	0	2	1		8		
	Female	1	1	1	1	0	3	0	2	1		10		
**Accuracy (n correct)^c^**														
	1a. Sad	2	2	2	1	1	4	—^d^	4	2		18	4.24 (0.75)	0
	1b. Happy	2	2	2	1	1	4	—	4	2		18	4.53 (0.80)	18
	2a. Sleep Poorly	1	1	0	1	1	1	—	1	2		8	3.65 (1.27)	4
	2b. Sleep Well	1	1	0	1	1	2	—	1	1		8	4.29 (0.85)	13
	3a. Worried	0	0	0	0	0	2	—	3	1		6	3.71 (1.05)	4
	3b. Not Worried	0	0	0	0	1	1	—	2	1		5	4.12 (0.86)	14

^a^Average rating of how well the animation captured the intended behavior (scale 1-5).

^b^Total number of children endorsing the animation as “more like them” from the respective pair.

^c^Number of children who correctly identified each animation.

^d^Accuracy responses unavailable for 10-year olds as no children of this age were recruited in this sample.

In terms of children’s ability to identify personal moments and/or construct examples that portrayed the target behaviors depicted by animations, some interesting findings were noted. For example, for the Worried and Not Worried pair of videos where fewer children initially identified the target behavior correctly, more children were able to provide examples that reflected scenarios where it might be appropriate to feel worried or not worried (confident). This suggests that though some children initially had difficulty either identifying or verbally expressing the targeted difficulty from the video (perhaps due to vocabulary limitations), their internal representation of these targeted difficulties may be more developed.

##### Understanding Instructions

Most children appeared to understand the question “which video is most like you” without further explanation. For example, children responded with comments such as “if you’re happy more days or not”; “what video I normally feel like”; and “what I’m feeling like most of the time.” Some younger children found it difficult to articulate a response to this question verbally, yet gave other nonverbal indications that they understood the question or were able to provide a response by pointing to one of the videos.

**Figure 3:**
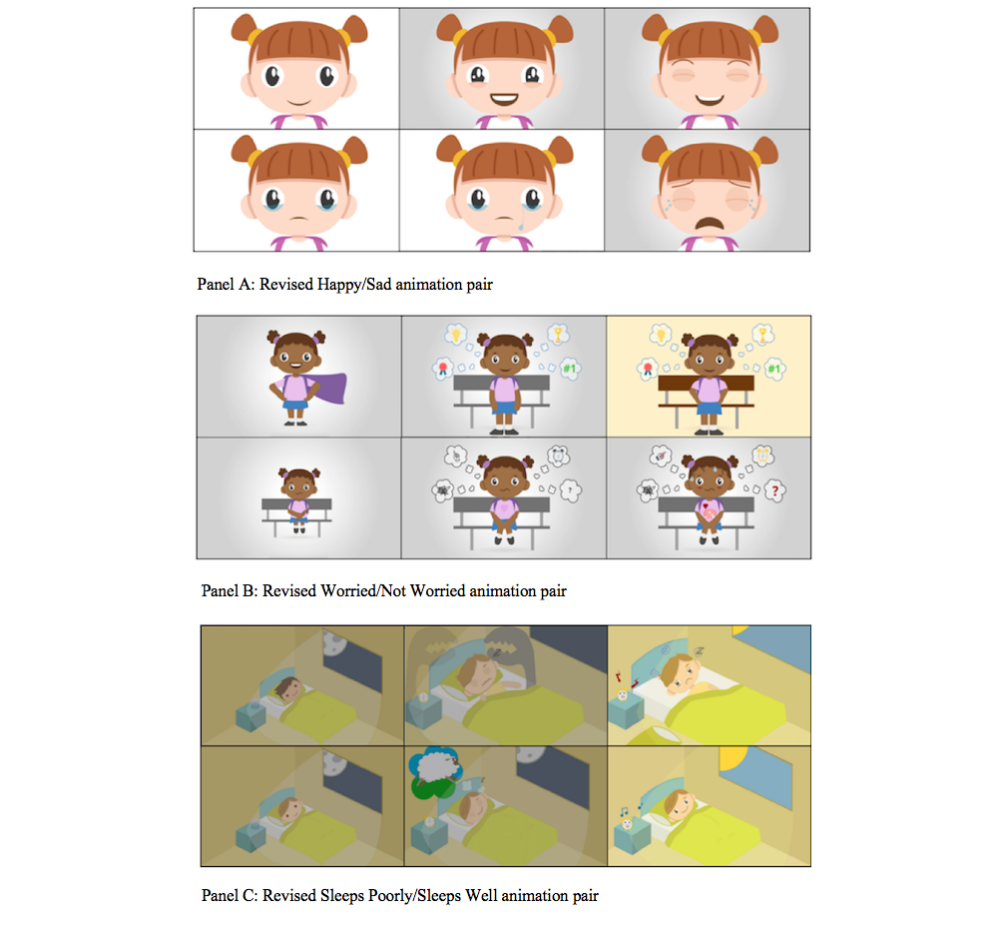
Screenshots showing refined pilot animations based on feedback from child interviews.

Of all children, 2 children (a 6 year old and an 11 year old) required some rephrasing of the question (eg, “which of these feelings do you feel most of the time?”) but then had no further difficulty. Only 1 child (a 4 year old) declined to choose a video from each pair that they thought was most like them. As outlined in Table 2, all children selected the positive video from the Happy or Sad pair, whereas 4 children selected the negative video from both the Sleeping and Worried or Confident video pairs. We note that this was a small sample of nonclinical children, so these figures are not considered representative of typical response patterns; nonetheless, they provide some indication that children may be willing to select the nonsocially desirable video when prompted to choose one or the other.

As expected, older children were more successful at articulating the difference between “a lot like you” and “a little like you.” Children provided responses such as “how often are you like that,” “how much are you like that feeling,” “is that how I am normally,” “do you have a little bit of that feeling in you or a lot,” and “are you always like this or only sometimes.” Of all responses, 2 responses (“when I feel the same emotions, I will show the same expression as the cartoon”; “how you look when you’re expressing that emotion—sometimes you be sad but you act happy”) were less accurate, 3 children did not respond to this question, and the youngest children (ie, less than 6) were more likely to repeat the language from the question, for example, “it means is she a little bit like me or not.” Overall, it appeared that despite variation in their ability to articulate a response verbally, most children (13/18; 72%) responded in a way that indicated a general understanding of the question. Nonetheless, it was clear that further practical testing would be beneficial in the context of the full app and with a larger sample.

##### Acceptability

Responses were overall positive regarding the general acceptability of the characters, animation style, and sounds. On the 5-point scale, the mean rating for likability of characters was 4.33 (SD 0.50) and for likeability of sounds was 4.13 (SD 0.64) out of 5. Children reported that they liked that the characters were colorful, pretty or “cute,” and enjoyed the variety of characters. The majority of children indicated that the sounds added to the videos made them easier to understand. Some children suggested adding more detail to the backgrounds to increase interest, which aligns with other suggestions that videos should include more context (eg, giving a speech in front of a class).

#### Step 4: Refinement of Animations

As a result of step 4, 3 new sets of animations were produced. Screenshots of the refined pilot animations are shown in Figure 3.

## Discussion

### Study Objectives

When identified early and appropriate interventions received, the adverse consequences of emotional and behavioral difficulties in childhood can be prevented. Universal screening has the potential to identify at-risk individuals likely to benefit from further assessment or intervention. To date, such screening instruments rely largely on parent, caregiver, or teacher report, despite evidence that children may be capable of providing valuable and accurate clinical information via self-report (eg, [13]). This paper sought to describe the development and piloting process for an animation-based screening instrument for early identification of childhood emotional and behavioral problems. Specifically, it described the initial development and feasibility testing stages of the ICDS, utilizing the mixed-methods approach. It is hoped that this study will provide insights to inform the development of future digital instruments for young people.

### Principal Findings

As a result of this study, we have identified 15 constructs or item groupings that will form the ICDS and that (1) are considered by experts as important for a broad emotional and behavioral distress screener, (2) are amenable to animation, (3) are distinct enough to warrant representation as a separate construct, and (4) incorporate items similar enough to plausibly tap into a global distress construct. This project further demonstrated that a child-focused, digital delivery interface and prototype animation items representing these constructs were acceptable to children and that children were able to accurately identify emotions and behaviors under the right conditions. Thus, the preliminary feasibility of the ICDS was demonstrated.

The findings of this project also demonstrate the utility of using mixed-methods approaches in the development of digital assessment tools. The results of study 1 demonstrated the benefit of involving an expert panel to identify and refine the constructs necessary for inclusion in a brief screening instrument, as well as in the identification of target constructs that could be best translated into an animated and child-report format. Given that such an instrument has not yet been developed, the inclusion of the expert panel allowed us to confirm the validity of the item selection and item groupings and prompted refinement of ICDS constructs. Specifically, this process allowed us to identify items that could be grouped together in one animation pair (eg, sad or unhappy) and others that were required to be captured independently (eg, fearful and worried). It also allowed us to confirm constructs and items that were of less importance in a broad screening instrument for childhood behavioral and emotional distress (eg, impulsive behavior, illicit and covert behavior). Expert review and iterative refinement in this way can be an important component in the development of new instruments, especially using new, innovative digital methods.

The second study sought to describe the development process at a step-by-step level to highlight the benefits of an iterative design and pilot testing in the end user group. Implementation of this method revealed a number of lessons regarding the development and use of digital animations in assessment tools for children. First, even at the first prototype stage, many children were able to understand the instructions and accurately identify the emotion or behavior being targeted in the animated items. This was more likely in target emotions and behaviors that are represented by clear external features such as sadness (tears) and sleeping difficulty (restlessness, looking tired) and less likely in complex emotions such as worry, where the emotion tends to be expressed inwardly (fearful thoughts, heart racing). The latter proved particularly challenging for younger children who may not understand labels such as “worry.” This highlights the careful consideration that must be given in translation of items into animated form and the necessity to review these with children of different ages. Although a strength of this study was its inclusion of a broad age range of youth, the small sample size within each age group requires these findings to be further examined in larger samples.

Second, findings suggested that even when children could not accurately label the target emotion or behavior, they were able to provide examples of similar behaviors or scenarios that suggested a more developed internal representation of these constructs. This reinforces the notion that using instruments that rely on a child’s cognitive and verbal ability to recognize and understand emotions may not provide reliable information. One benefit of a digital assessment tool such as the ICDS may be that concepts difficult for children to understand using verbal or written approaches may be more easily communicated through animations. In the ICDS, children merely need to recognize or relate to one of the visually depicted response options; this approach could be far easier than written descriptors (eg, I worry a lot) that may be too abstract and complicated to understand.

Third, findings from this development and testing process indicated that children could understand the instructions and requirement to choose which animation was more like them; however, older children were better at articulating the difference between levels of likeness (eg, “a lot like you” or “a little like you”). Fourth, the importance of using colorful and simple images and design was confirmed through participant acceptability and feedback. Such findings are not dissimilar to those in the child eHealth literature, which demonstrate the effectiveness of interventions that utilize eye-catching graphics, colors, stories, animations, and interactive activities [35-37]. Fifth, the importance of depicting strong, highly visible displays of the target problem was noted by many young people as a strategy for more clearly helping children to understand the target emotion or behavior. This may indicate that children find it difficult to identify the behavior or emotion at lower, more subtle levels of intensity.

Finally, children frequently cited the need to contextualize animations to achieve accurate understanding of target items. Contextualizing animated items presents both potential benefit and difficulty. Traditional pen-and-paper screening instruments typically remain vague and overly general in their item descriptions (eg, “I feel sad” or “I tend to worry about things”) so as not to imply a problem specific only to a certain context (eg, only at school, with parents). Given the known heterogeneity in which emotional and behavioral problems may manifest in children (eg, [38]), it is necessary to ensure that as many different representations of the target problem are represented as possible. We deliberately developed our prototype animations with sparse backgrounds to encourage children to focus on the general behavior or feeling being targeted, rather than associating it with a specific activity or context; yet child feedback suggests that this may be necessary in digital tools. Our refined animations addressed this by incorporating contextual information, for example, a park background in our worried or confident item, without adding elements that may be associated with specific forms of worrying such as a dog or other children. Assessing the match between animations and intended item meaning will be crucial to the development of future digital instruments for children.

### Implications and Future Research

The ICDS is intended for use as a screening instrument that can assist families and relevant professionals (eg, family health practitioners, teachers) to identify potential difficulties and guide decision making around referrals to formal assessment or interventions. The results of this project are being used to guide and inform the development of the remaining ICDS items, which will also be developed using an iterative codesign process. Future studies will confirm the utility of the ICDS in detecting childhood emotional and behavioral distress compared with existing child- and parent-report instruments in community and clinical samples. This research will also allow the identification of “at-risk” cut-offs for different groups, which is necessary before widespread dissemination can occur. If effective, the ICDS will present a screening instrument that may be highly accepted by young people and provide valuable child clinical reports to inform further assessments and intervention referrals. An additional benefit of using modern Web-based technologies is the possibility of future over-the-air updates, allowing ongoing development to remain responsive to user feedback. Further, no specialist equipment or training will be necessary for the ICDS, thus providing a screening instrument that is easily disseminated and can have maximum prevention and early intervention capacity. Although this prototype has been developed only for English-speaking youth, future versions may be contextualized for other languages, as well as for specific emotional and behavioral disorders.

### Summary

This project described the development of an animated screening instrument for childhood emotional and behavioral distress, reporting on results of expert panel review and refinement of constructs, as well as pilot testing with children. The mixed-methods approach to development and testing revealed valuable information from experts and the target child group that assisted in the iterative refinement of the screener. The ICDS has potential to obtain clinical information from the child’s perspective, which may be missed through other observer report. There are very few child-reported screening instruments available for use, and if effective, the ICDS will provide a quick, engaging, and easy-to-use screener that can be utilized by families and in routine care settings. This project highlights the importance of involving expert review and user codesign in the development of digital assessments for children.

## Appendix

Multimedia Appendix 1 Preliminary and revised construct groupings, utilizing items from three brief measures of child emotional and behavioral difficulties.

Multimedia Appendix 2 Verbatim feedback from children (by age and gender), including initial interpretation of items and whether considered correct by the interviewer; suggestions from child for improving animations to better convey intended meaning; and child’s personal story, identifying a time when they have felt like the character in the animation.

## Abbreviations

BPM: Brief Problem Monitor
CBCL: Child Behavior Checklist
ICDS: Interactive Child Distress Screener
M&MS: Me and My School Questionnaire
SDQ: Strengths and Difficulties Questionnaire
SQL: structured query language
