# Supplementary material for: The Interactive Child Distress Screener: Development and Preliminary Feasibility Testing
Source: JMIR Mhealth Uhealth. 2018 Apr 19;6(4):e90. doi: 10.2196/mhealth.9456 (PMC5934532; doi:10.2196/mhealth.9456)
Supplement: Multimedia Appendix 1 [file mhealth_v6i4e90_app1.pdf]

Initial domain	Example items	Scale	Revised domain	Example items	Scale
1. Nervous/shy	I am shy	M	1. Shy	I am shy	M
	Nervous or clingy in new situations, easily loses confidence	S		Self-conscious or easily embarrassed	B
	Self-conscious or easily embarrassed	B			
2. Sad/depressed	I am unhappy	M	2. Sad/depressed	I am unhappy	M
	Often unhappy, depressed or tearful	S		Often unhappy, depressed or tearful	S
	I cry a lot	M		I cry a lot	M
	Unhappy, sad, or depressed	B		Unhappy, sad, or depressed	B
	Feels worthless or inferior	B			
3. Worried/anxious	I worry when I am at school	M	3. Worried	I worry a lot	M
	I worry a lot	M		Many worries or often seems worried	S
	Many worries or often seems worried	S		Worries	B
	Worries	B			
	Often complains of headaches, stomach-aches or sickness	S	4. Physical symptoms	Often complains of headaches, stomach-aches or sickness	S
	Too fearful or anxious	B	5. Fearful	Too fearful or anxious	B
	Many fears, easily scared	S		Many fears, easily scared	S
	I feel scared	M		I feel scared	M
4. Sleep problems	I have problems sleeping	M	6. Sleep problems	I have problems sleeping	M
	I wake up in the night	M		I wake up in the night	M
5. Irritable, argumentative, easily loses temper	I get very angry	M	7. Angry	I get very angry	M
	I lose my temper	M		I lose my temper	M
	Temper tantrums or hot temper	B		Temper tantrums or hot temper	B
	Often loses temper	S		Often loses temper	S
	I am calm (+)	M			
	Stubborn, sullen, or irritable	B			
	Argues a lot	B	8. Argumentative	Argues a lot	B
6. Aggressive behavior	Often fights with other children or bullies them	S	9. Physically aggressive	Often fights with other children or bullies them	S
	I do things to hurt people	M		I hit out when I am angry	M
	Threatens people	B		I break things on purpose	M
	I hit out when I am angry	M		Destroys things belonging to his/her family or others	B
7. Destructive behavior	I break things on purpose	M		I do things to hurt people	M
	Destroys things belonging to his/her family or others	B			
8. Social problems	I feel lonely	M	10. Difficulty making friends	I feel lonely	M

Initial domain	Example items	Scale	Revised domain	Example items	Scale
	Nobody likes me	M		Nobody likes me	M
	Has at least one good friend (+)	S		Has at least one good friend (+)	S
	Gets along better with adults than with other children	S			
	Picked on or bullied by other children	S	11. Bullied or teased by other children	Picked on or bullied by other children	S
	Generally liked by other children (+)	S		Generally liked by other children (+)	S
	Rather solitary, prefers to play alone	S			
9. Noncompliant behavior	Disobedient at school	B	12. General noncompliance: School context	Disobedient at school	B
	Disobedient at home	B	13. General noncompliance: Home context	Disobedient at home	B
	Generally well behaved, usually does what adults request (+)	S			
10. Illicit or covert behavior	Steals from home, school or elsewhere	S			
	Often lies or cheats	S			
	Feels too guilty	B			
11. Impulsive behavior	Impulsive or acts without thinking	B			
	Thinks things out before acting	S			
	Acts too young for his/her age	B			
12. Inattentive behavior	Inattentive or easily distracted	B	14. Inattentive behavior	Inattentive or easily distracted	B
	Easily distracted, concentration wanders	S		Easily distracted, concentration wanders	S
	Fails to finish things he/she started	B		Can’t concentrate, can’t pay attention for long	B
	Can’t concentrate, can’t pay attention for long	B		Good attention span, sees chores or homework through to the end	S
	Good attention span, sees chores or homework through to the end	S			
13. Hyperactive behavior	Can’t sit still, restless or hyperactive	B	15. Hyperactive behavior	Can’t sit still, restless or hyperactive	B
	Constantly fidgeting or squirming	S		Constantly fidgeting or squirming	S
	Restless, overactive, cannot stay still for long	S		Restless, overactive, cannot stay still for long	S
14. Helpful and considerate of others (+)	Kind to younger children	S			
	Shares readily with other children, for example toys, treats, pencils	S			
	Considerate of other people’s feelings	S			
	Helpful if someone is hurt, upset or feeling ill	S			
	Often volunteers to help others (parents,	S			

Initial domain	Example items	Scale	Revised domain	Example items	Scale
	teachers, other children)				

Note. (+) Item or domain is worded positively, reflecting an absence or lack of difficulty in that domain. S = Strengths and Difficulties Questionnaire [25]; B = Brief Problem Monitor [29]; M = Me and My School Questionnaire [30].
