# Supplementary material for: The Interactive Child Distress Screener: Development and Preliminary Feasibility Testing
Source: JMIR Mhealth Uhealth. 2018 Apr 19;6(4):e90. doi: 10.2196/mhealth.9456 (PMC5934532; doi:10.2196/mhealth.9456)
Supplement: Multimedia Appendix 2 [file mhealth_v6i4e90_app2.pdf]

Video	Age	Gender	Interpretation	Correct?	How improve?	Personal story
Sad	4	M	Sad	Y	Like that (points to target cartoon)	When no one wants to play with me, even my sister
	4	F	Sad	Y	-	When Mummy was dropping me off at kindy
	5	M	Sad	Y	-	When [brother] tripped over and hurt his hand and foot and he was sad
	5	F	Sad	Y	Show a bully	-
	6	M	Sad	Y	Show somebody crying	-
	6	F	Sad	Y	Show him crying more	When my friend didn't get to kick the ball
	7	F	Sad	Y	-	When she got a red ant bite
	8	M	Sad	Y	A bit more actions	When no-one wants to play with me
	9	M	Sad	Y	-	When you're left out of games at school
	9	F	Sad	Y	-	She was getting bullied
	9	F	Sad	Y	-	At home when my brother was sad
	9	F	Sad	Y	-	When I had to move to a new school and leave my friends
	11	M	Sad and crying	Y	Show a kid dropping ice-cream on the floor and then crying	When our cat Chilli died
	11	M	Crying. Sad, worried, stressed	Y	Bit more action, show what she's sad about	-
	11	F	Sad	Y	-	When I was getting bullied
	11	F	Sad	Y	-	-
	12	M	Upset	Y	Have friends running, show him more upset	-
	12	F	Sad	Y	Show someone getting upset	When a pet died
Happy	4	M	Happy	Y	Like that (points to target cartoon)	When Daddy was singing songs with me
	4	F	Happy	Y	-	When I was going to the Despicable 3 movie
	5	M	Happy	Y	-	When my friend was playing with me
	5	F	Happy	Y	I stand up to the bullies, I stand up for myself	-
	6	M	Happy	Y	Show somebody shouting out 'Yay' and jumping	-
	6	F	Happy	Y	Show his bottom teeth	When I get to play with my friend
	7	F	Happy	Y	-	When she didn't need to be the Mum in the games all the time
	8	M	Really happy	Y	A little more actions too	When I've done something good and feel proud
	9	M	Happy	Y	-	When you feel not left out
	9	F	Happy	Y	-	She met new friends
	9	F	Happy	Y	-	When we got our cat
	9	F	Happy	Y	Jumping up and down and looking excited	When I got my NAPLAN results – I did well

Video	Age	Gender	Interpretation	Correct?	How improve?	Personal story
	11	M	Happy	Y	-	When I got my BMX bike
	11	M	Happy, did well	Y	Same but for happy	-
	11	F	Happy	Y	-	When I found out about a holiday
	11	F	Happy	Y	-	All the time
	12	M	Happy	Y	Having fun on a playground	-
	12	F	Happy	Y	Someone who won something, smiling joyfully	When I won a medal
Sleeping poorly	4	M	Having a bad sleep	Y	Like that (points to target cartoon)	When I woke up at kindy I had a bad dream of monsters and wolves eating me
	4	F	Sad in his bed		-	Because I had a bad dream
	5	M	Tired and sleepy		-	There was a scary noise and I couldn't sleep so I went to Mum's room
	5	F	Not happy, he didn't have a good sleep, not comfortable	Y	-	-
	6	M	Sleepy		-	-
	6	F	Sad and tired		Take the moon away	-
	7	F	Boy didn't get enough sleep	Y	-	When I stay up too late
	8	M	Couldn't sleep well, feels really tired	Y	Add a dream bubble	I have nightmares a lot about things eating my brains
	9	M	Tired and sad		Show him get out of bed and walk like a zombie	I fell out of bed
	9	F	Uncomfortable		Nightmare dream bubble above his head	-
	9	F	Uncomfortable		Show him a bit more cranky	Bad dreams make me sleep bad
	9	F	Slept badly	Y	-	At my friend's house sleepover, it was unfamiliar
	11	M	Moving in their sleep, seems angry		Show someone getting out of bed in the morning, hunched over, really tired, dragging their feet, yawning	-
	11	M	Can't sleep, had nightmare, really tired	Y	-	-
	11	F	Uncomfortable		Add a nightmare dream bubble; get out of bed looking sleepy and walking slowly	If I have a nightmare or I can't sleep
	11	F	Uncomfortable		-	Tired and grumpy
	12	M	Trouble sleeping	Y	-	-
	12	F	Can't get to sleep	Y	-	When you feel bad for doing something good
	4	M	Having a good sleep	Y	Like that (points to target cartoon)	My happy dream was I went to a tea party

Video	Age	Gender	Interpretation	Correct?	How improve?	Personal story
Sleeping well	4	F	Happy in his bed		-	Because I had a good sleep
	5	M	Sleepy and wakey		-	-
	5	F	He had a good sleep	Y	-	-
	6	M	Sleepy		-	-
	6	F	Okay		Make the window somewhere else	-
	7	F	He got enough sleep	Y	-	When I go to bed early
	8	M	He had a nice sleep	Y	Add a dream bubble	After a fun day and I have good dreams
	9	M	Happy		Show him jump out of bed in the morning energetic	Wake up happy
	9	F	Tired		Happy dream bubble above his head	-
	9	F	He had a good night	Y	Show yawning and smiling	-
	9	F	Slept well	Y	-	When I have a late night the day before
	11	M	He came back from the dead, he did not move		Show someone jumping out of bed with lots of energy and smiling	After a sleep-in, when you don't have to wake up early
	11	M	Didn't dream or had good dreams	Y	-	-
	11	F	Sleepy		Add a happy dream bubble	-
	11	F	Comfortable		-	When I feel rested and good
	12	M	Good night's sleep	Y	-	-
	12	F	Tired		-	When you've had a busy day and are really tired
Worried	4	M	Angry – hurt her hand		Like that (points to target cartoon)	I was on the stage at kindy and I didn't know what we were doing
	4	F	Don't know – sad and lost		-	When I wanted my Mummy
	5	M	Angry and sad		-	Sometimes I worry because my friend wasn't playing with me
	5	F	Hungry		-	-
	6	M	Sad		She can't find her Mum and Dad	-
	6	F	Just a bit sad		Make her smile	-
	7	F	Bored		Doing a test in class and a very worried face	-
	8	M	Bit sad and lonely		A more scared face, more actions	When I think I've lost Mum and Dad in the shops
	9	M	Sad		-	When my friend got lost
	9	F	Confused		She could be looking around more	-
	9	F	Nervous	Y	-	Feel a bit sick like butterflies in my tummy
	9	F	Nervous – same as worried	Y	--	Backstage before my dance concert

Video	Age	Gender	Interpretation	Correct?	How improve?	Personal story
	11	M	Alone and anxious waiting at a bus stop	Y	A student receiving test results, waiting for test results	Doing NAPLAN
	11	M	Anxious, worried, waiting	Y	Show what she's worried about – add a bus stop and a clock to show it's late	-
	11	F	Waiting and lonely		More thought bubbles of worrying things	When I couldn't find my mum
	11	F	Nervous	Y	-	Getting to school late
	12	M	Lonely		-	-
	12	F	Worried	Y	Someone taking a test	About exams
Confident	4	M	Happy		Like that (points to target cartoon)	When I was playing a superhero and I helped them pack up the mess
	4	F	Happy		-	-
	5	M	Happy		Talking to all the kids at kindy	When I was talking out at my kindy – when I was doing my show-and-tell of the Eiffel Tower when I see'd it and took a picture of me in front of the Eiffel Tower
	5	F	Happy		-	-
	6	M	Happy		-	-
	6	F	Happy and okay		Show some friends around her sitting beautifully listening to her talk	When I'm dancing in a concert
	7	F	Happy		Getting a high score in a maths test	My brother is confident
	8	M	Confident and proud	Y	-	-
	9	M	Really happy		-	-
	9	F	Feels like a superhero; energetic		Throw the cape off	When my friend was doing show and tell and had memorised her speech
	9	F	Excited		-	-
	9	F	Confident	Y	-	I knew my part really well in speech and drama
	11	M	Confident	Y	Someone doing a presentation in class, with no palm cards and smiling, standing up straight	When I was doing a presentation and I knew everything I had to say
	11	M	Role playing, feels good about himself		-	-
	11	F	Special and proud		Have a medal pop up on her chest or trophy in her hand; or singing in front of friends	For my first singing and guitar gig
	11	F	Confident	Y	-	When I hand in assignments on time
	12	M	Brave	Y	-	-

Video	Age	Gender	Interpretation	Correct?	How improve?	Personal story
	12	F	Happy		Someone doing a talk or presentation in front of the class with confidence	In front of the class
